# Intensification of strontium (II) ion biosorption on *Sargassum sp* via response surface methodology

**DOI:** 10.1038/s41598-023-32532-5

**Published:** 2023-04-03

**Authors:** F. Soleymani, M. H. Khani, H. Pahlevanzadeh, Younes Amini

**Affiliations:** 1 Chemical Engineering Department, Tarbiat Modares University, P.O. Box 14155-143, Tehran, Iran; 2 Nuclear Fuel Cycle Research School, Nuclear Science and Technology Research Institute, P.O. Box 11365-8486, Tehran, Iran

**Keywords:** Biochemistry, Chemical engineering

## Abstract

A batch system was employed to investigate the biosorption of strontium (II) on *Sargassum sp.* The biosorption of strontium on *Sargassum sp* was studied with response surface methodology to determine the combined effect of temperature, initial metal ion concentration, biomass treatment, biosorbent dosage and pH. Under optimal conditions, the algae's biosorption capacity for strontium (initial pH 7.2, initial strontium concentration 300 mg/l for Mg-treated biomass and biosorbent dosage 0.1 g in 100 mL metal solution) was measured at 103.95 mg/g. In our analysis, equilibrium data were fitted to Langmuir and Freundlich isotherms. Results show that the best fit is provided by the Freundlich model. Biosorption dynamics analysis of the experimental data indicated that strontium (II) was absorbed into algal biomass in accordance with the pseudo-second-order kinetics model well.

## Introduction

Keeping radioactive substances contained is a crucial safety issue for nuclear industries^1–6^. The beta-emitting strontium (Sr)-90 radionuclide has a half-life of approximately 28 years, and it is produced by nuclear fission reactions. Water and wastewater industries use adsorption as one of the most important separation and purification processes to remove color, odor, and organic pollution^7–13^. There is a wide variety of microorganisms including bacteria, fungi, and algae that are used as metal adsorbents^14–19^. Also, the different synthesis procedures using biopolymer namely chitosan were applied to the adsorption of strontium from aqueous solutions^20–25^. Wang et al.^26^ used a carboxyl functionalized resin, i.e. SiMaC, prepared with in situ polymerization to adsorb strontium. They observed the maximum capacity of 142.5 mg/g at room temperature and fitting adsorption isotherms well with a pseudo-second-order (PSO) kinetics model. Qiu and co-workers^27^ studied the adsorption of strontium by living Saccharomyces cerevisiae. They concluded that bioaccumulation is vital during biosorption, and ion exchange is the main mechanism during separation compared to physical adsorption.

Among these microorganisms, Marine algae are more popular due to several parameters like low cost, their high metal biosorption capacity and their renewable nature^28^. Algal cell walls contain functional groups such as carboxyl, hydroxyl, and sulfhydryl, which allow them to attach metal ions from water^29^. Prabhu et al.^30^ studied the capability of the pteridophyte on the adsorption of Pb(II) from an aqueous solution. The biosorption capacity of 125 mg/g was observed with a removal efficiency of 99.93%. Yue and co-workers^31^ adsorbed Cd(II), Pb(II), and Cu(II) from an aqueous solution by using the amidoxime functionalized cellulose-based adsorbent. They observed that the Freundlich model predicted well the adsorption isotherm of both Cu(II) and Pb(II) ions, while the Langmuir model fitted well the adsorption isotherm of Cd(II).

As a tool, response surface methodology (RSM) can be active to investigate the interactions among two or more factors. Statistical and mathematical techniques are used in RSM to improve and optimize processes. In general, it involves three stages: (i) design and experiments, (ii) regression modeling, and (iii) optimization^32–37^.

In this study, *Sargassum sp* and chemically modified *Sargassum sp* were used for their ability to remove Sr from the solution. A feasibility study is conducted to investigate the use of adsorbent to remove Sr^+^^2^ from aqueous solutions by varying pH, temperature, initial strontium concentration, and brown algae dosage. We use the central composite design method to serve this purpose.

## Materials and methods

### Preparation of biosorbents

We collected samples of the marine algae Sargassum sp in the Persian Gulf along the coast of Qeshm, Iran. A combination of tap distilled water and water was used to wash the algae samples. This was done in order to remove sand as well as excess sodium and potassium ions. The samples were dried overnight at a maximum temperature of 55 °C, then ground to an average particle size of 0.7 mm to avoid degradation of binding sites^38^. Following loading with Mg2 + , the biomasses were soaked in 0.1 M Mg(NO_3_)_2_·6H_2_O (biomass concentration, 10 g/l) for 24 h under slow stirring. It was then necessary to wash the pretreated biomass several times with deionized water to reach a stable pH level for the washing solution, as well as to remove excess ions of magnesium from the biomasses. To use the Mg-pretreated biomasses in biosorption experiments, the biomasses were first dried overnight in an oven at 55 °C, and then natural algae as well as Mg-pretreated algae were utilized for the biosorption experiments.

### Preparation of metal solutions

Strontium stock solutions containing 1000 ppm of strontium were prepared using analytical grade Sr(NO_3_)_2_ and the concentration of this solution was diluted based on the requirements. It should be noted that all solutions were prepared using deionized water. A dilute or concentrated solution of HNO_3_ and NaOH was used to adjust the pH of the strontium solutions before mixing them with the biosorbent. A grade of analytical chemicals was used throughout the experiment (Sigma Aldrich, Germany).

### Analytical procedures

Energy dispersive X-ray spectroscopy (EDX, RONTECH, Germany) was utilized to analyze the properties of biosorbent earlier and subsequently its treatment with 0.1 M Mg (NO_3_)_2_·6 H_2_O and subsequently Sr^2^^+^ sorption.

A spectrophotometer based on atomic emission spectroscopy (ICP-AES, Optima, 7300DV, USA) and inductively coupled plasma were utilized to measure the levels of strontium dissolved in the solution. In this case, the ICP analysis was investigated at a wavelength of 460.733 nm.

### Batch adsorption experiments

We performed sorption dynamics experiments on naturally occurring algae and on Mg-pretreated algae first to obtain the required contact time of sorption equilibrium experiments. In 250 ml Erlenmeyer flasks including 100 ml of 100 mg Sr/l Sr(NO_3_)_2_ solution, 0.1 gr of biomass was added. A shaker was used to agitate the flasks at 150 rpm and 25 °C for 24 h. A representative sample was selected at predetermined intervals over a period of time (2, 5, 15, 30, 45, 60, 90, 120, 150, 180 and 1440 min). ICP-AES spectrometry was then used to analyze the samples after dilution to determine the metal concentrations. It has been observed that the equilibrium of sorption dynamic tests was achieved after 150 min of contact. In batch adsorption experiments, magnesium ions were studied as surface modifiers of temperature, pH, biomass, biomass dosage and strontium concentration. A 250 ml Erlenmeyer flask containing 100 mL of Sr(NO_3_)_2_ solution with an initial concentration of strontium with different amounts of biomass was mixed. It is important to note that the flasks were shaken at a speed of 150 revolutions per minute for a period of 150 min. A diluted or concentrated solution of NaOH and HNO_3_ was mixed with the initial solution to adjust the pH before adding the biosorbent. In order to filter the strontium solution, Whatman filter paper was used. Following the filtering of the samples, the residual strontium ion concentration was measured. Comparing the amount of metal added to the biomass with the amount of metal in the supernatant, we calculated the amount of metal taken up by algae using Eq. 1:

1 $$q = \frac{{V(C_{0} - C)}}{{M_{ads} }}$$

In this equation, q (mg/g) at any given time t (min) is the adsorbed metal ions on the biosorbent, V (ml) is the volume of the metal-containing solution that is coming into contact with the biosorbent, C_0_ (mg/l) is the metal initial concentration, C (mg/l) at any given time t (min) is the concentration of metal in the solution, and M_ads_ (g) is added biosorbent^39^.

### Experimental design of biosorption testing

An experimental design was utilized to examine the effects of four numerical factors (biosorbent dosage, initial pH, initial metal ion concentration and temperature) at 5 levels and one categorical factor (biomass treatment) at 2 levels on the biosorption of strontium on *sargassum sp* and obtain optimal conditions. The Design-Expert software (version 7.1.4) was applied (Table 1) as part of the experimental design, All through testing, α was supposed to be 2.

**Table 1:** The experimental range and levels of the control factors in the CCD.

Variable	Low axial (− α)	Low factorial (− 1)	Center	High factorial (+ 1)	High axial (+ α)
*x*_1_(A): Temperature (^°^C)	15	23	30	38	45
*x*_2_(B): Initial strontium (II) concentration (mg/l)	50	113	175	238	300
*x*_3_(C): Biomass dose (g/100 ml)	0.1	0.2	0.3	0.4	0.5
*x*_4_(D): pH	2.5	5.1	7.8	10.4	13
	Level 1		Level 2		
*x*_5_(E): Type of biosorbent	natural *Sargassum sp.*		Mg- pretreated *Sargassum sp.*		

*α = 2.

To select the range of studied variables in Table 1, the temperature range was chosen to be around the ambient temperature with ± 15 due to proximity to possible industrial operating conditions. To investigate the effect of initial concentration, the range of real waste concentration and its simulation were considered. The widest range was chosen to investigate the effect of pH due to a lack of primary sense. To investigate the dose effect, the author’s previous experiences regarding biosorption processes were considered^40–47^.

Several techniques are applied to fit the polynomial model, including the central composite design (CCD)^48–50^. As a result of this method, as the mathematical model is empirical, modelling is simplified and the detailed reaction mechanism does not need to be known. In CCD, the number of trials is calculated as k + 2 k + n, where k represents the number of independent variables, 2 k represents the number of axial points, and n represents the number of center points^51–56^.

A linear or quadratic model can be used to relate the responses to the chosen factors in the optimization process. The quadratic model, along with the linear model, is written as;

2 $$y = b_{0} + \sum\limits_{i = 1}^{k} {b_{i} } x_{i} + \sum\limits_{i = 1}^{k} {b_{ii} x_{i}^{2} } + \sum\limits_{i = 1}^{k} {\sum\limits_{j = 1}^{k} {b_{ij} x_{i} x_{j} } }$$

where y is the response variable’s expected value; *b*_*0*_*, b*_*i*_*, b*_*ii*_*, b*_*ij*_ are the model parameters; and x_j_ and x_i_ are the factors. For the empirical models, y1 and y2 represent the biosorption capacities of natural and modified algae, respectively, for the empirical models.

### Confirmation experiment

A confirmatory experiment was conducted to check whether the models were valid by setting the experimental values at optimal levels expected by the models. Comparing biosorption capacity values obtained from the experiment with predictions from models, we checked whether they were within low or high confidence intervals.

## Results and discussion

### Statistical analysis

We used analysis of variance (ANOVA) results (see Table 2) to study the effect of different factors, e.g. biosorbent dosage, biomass treatment temperature, pH and metal ion concentration, on the response of the system. It is necessary to use this statistical device in order to examine the adequacy and significance of the model. As a measure of the degree to which the factors attempt to describe the variation in the data about the means, the Fischer variation ratio (F-value) can be considered. Analysis of variance F-values is calculated by dividing MS caused by model variation by MS caused by variance in error. As data have some variation around their mean value, it is generally accepted that the greater the F-value from the unity the more variation is the most acceptable^57–59^. Ideally, the F-value should be several times higher than the tabulated value. Additionally, *p* values less than 0.05 point to significant model terms. A *P*-value of less than 0.05 implies that the biosorption capacity model is statistically significant, as shown in Table 2. According to Table 2, the temperature is not significantly different from other factors such as CE (Mads, Type of biosorbent) and BE (C0, Type of biosorbent).

**Table 2:** Analysis of variance for the response surface reduced quadratic model for strontium biosorption.

Source	Sum of squares	Mean square	df	*p*-value	F value
Model	13,174.70	1463.86	9	< 0.0001	307.81
A-T	5.23	5.23	1	0.3032	1.09
B-C_0_	6213.48	6213.48	1	< 0.0001	1306.53
C-Mads	5651.55	5651.55	1	< 0.0001	1188.37
D-pH	35.74	35.74	1	0.0087	7.52
E-E	258.52	258.52	1	< 0.0001	54.36
BE	46.85	46.85	1	0.0030	9.85
CE	46.45	46.45	1	0.0031	9.77
B^2^	122.46	122.46	1	< 0.0001	25.75
C^2^	394.54	394.54	1	< 0.0001	82.96
D^2^	226.26	226.26	1	< 0.0001	47.58
Residual	218.76	4.76	46		
Lack of Fit	217.89	5.45	40	< 0.0001	37.47
Pure Error	0.87	0.15	6		
Cor Total	13,393.46		55		

### Fitting models

A CCD design was used to analyze 56 runs to obtain biosorption capacity. A modified quadratic model polynomial equation was fitted to the experimental results of the CCD using multiple regression analysis. Based on Eqs. (3) and (4), we calculate the biosorption capacity for strontium (II) ions in untreated and treated biomasses, respectively.

3 $$q_{1} = 41.60719 + 0.30481x_{2} - 265.22708x_{3} + 4.4x_{4} - 004x_{2}^{2} + 277.59375x_{3}^{2} - 0.30508x_{4}^{2}$$

4 $$q_{2} = 46.2745 + 0.33642x_{2} - 284.90208x_{3} + 4.4x_{4} - 004x_{2}^{2} + 277.59375x_{3}^{2} - 0.30508x_{4}^{2}$$

**Figure 1:**
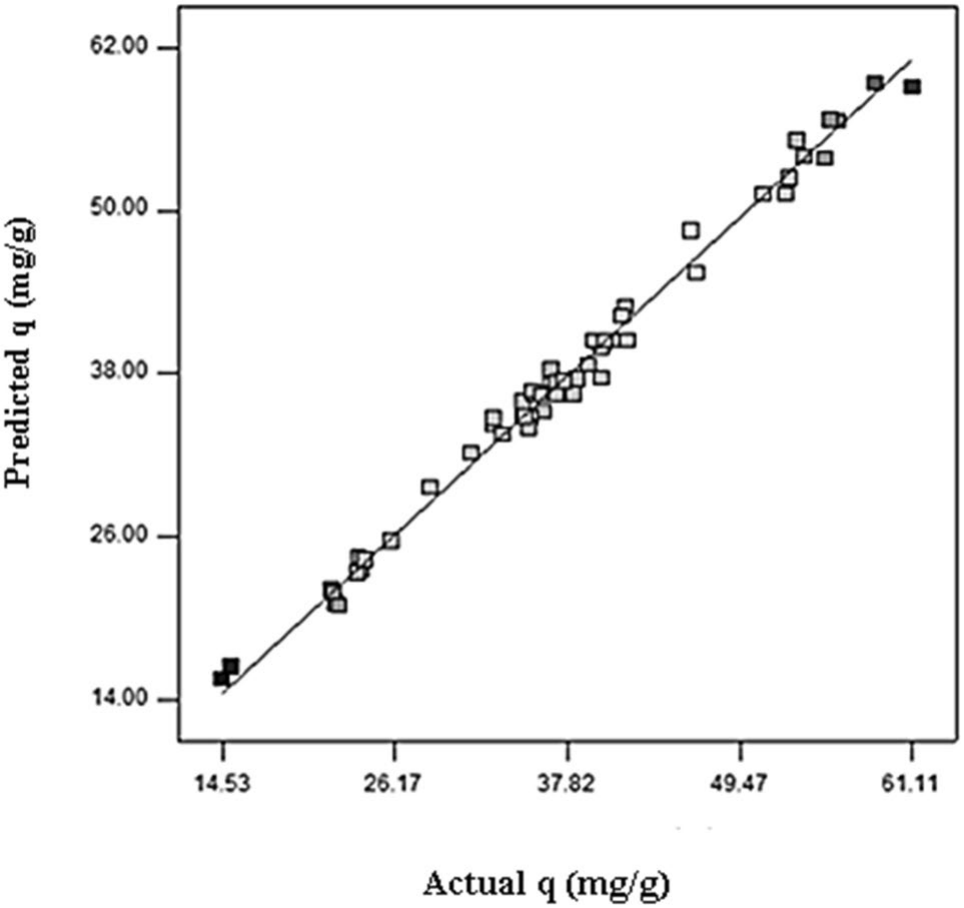
Scatter graph of the predicted response values versus the actual response values for the biosorption capacity of biomass for strontium.

**Figure 2:**
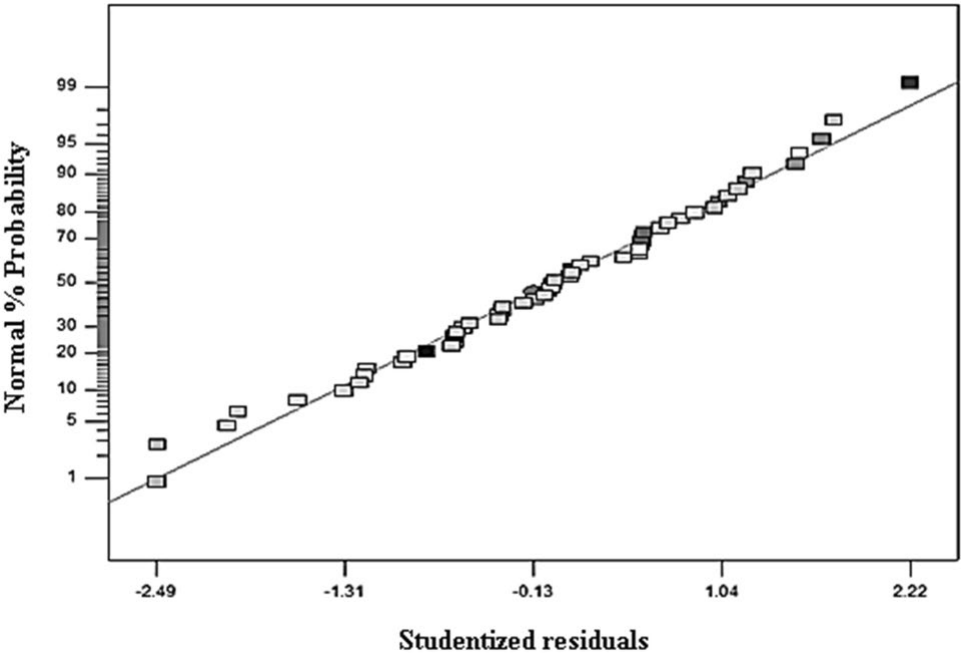
Normal plot of studentized residuals versus normal % probability for bearing the experiment for strontium ions.

The above equations express the effects of various operating parameters such as pH, initial pollutant concentration, adsorbent dose, and also their interference effects in the adsorption rate of the studied adsorbents. We applied the polynomial models which could be considered as an approximation of the true functional relationship over relatively small regions of an independent variable space^60^. The predicted data as a function of actual data obtained from the experimental results is depicted in Fig. 1. A satisfactory correlation can be found between experimental data and predicted values based on the clustering around the diagonal line, which confirms the robustness of the model. As can be seen in Table 3, the modified quadratic model for biosorption capacity has high R^2^, adjusted R^2^, and adjusted R^2^ adj numbers, indicating that it can accurately represent the system under the given experimental conditions. To have adequate precision, it is important that the signal-to-noise ratio be higher than four, which is desirable. An increase in CV over 10 indicates a high variation in mean value, which prevents a satisfactory response model from developing^32^. Table 3 contains data that confirms these results. In Fig. 2, we show a normal probability plot and a studentized residual plot for the biosorption capacity of *sargassum sp* for strontium (II) ions. In addition to this, the residuals provide insight into the model's compliance with ANOVA assumptions, where the studentized residuals measure the difference between the predicted and actual values.

**Table 3:** Statistical results of the ANOVA for the reduced quadratic model.

R^2^	0.9837
C.V %	4.80
Adjusted R^2^	0.9805
Adequate precision	71.658

### Process optimization using desirability functions

During the process, strontium (II) ions were absorbed by the biosorbent by maximizing biosorption capacity and finding optimal conditions. There are minimum and maximum levels that need to be specified in numerical optimization for each parameter. Multiple starting points in the design space section could highly improve the success rate of finding the best local maximum^61^. A 300 mg/l strontium (II) concentration, 0.1 g in 100 mL of biosorbent, and 7.2 pH were estimated to be the optimum values, respectively. The temperature does not affect optimization results, as shown in Table 2. Biosorbents with Mg(NO_3_)_2_·6H_2_O-treated biomass had the most metal uptake (103.95 mg/g) under optimum conditions (Mg-treated biomass).

### Effect of Mg^2+^ as a pretreatment on biosorption capacity

**Figure 3:**
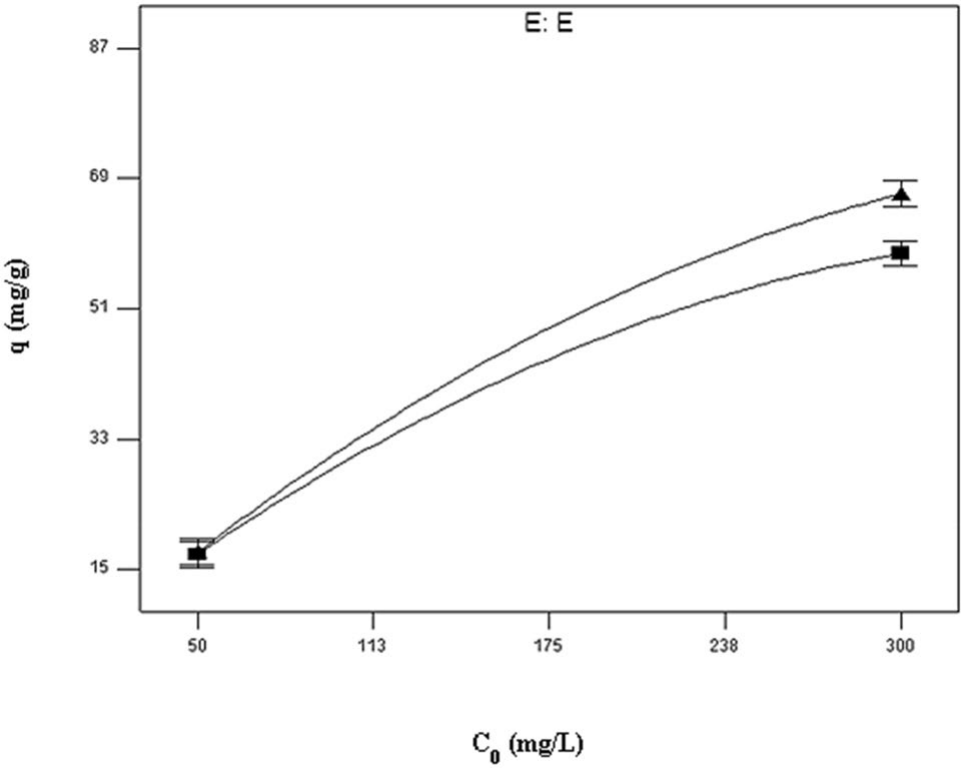
Interaction plot showing the effect of type of biomass and initial strontium (II) concentration on the strontium uptake of biomass holding other variables at their central values.

**Figure 4:**
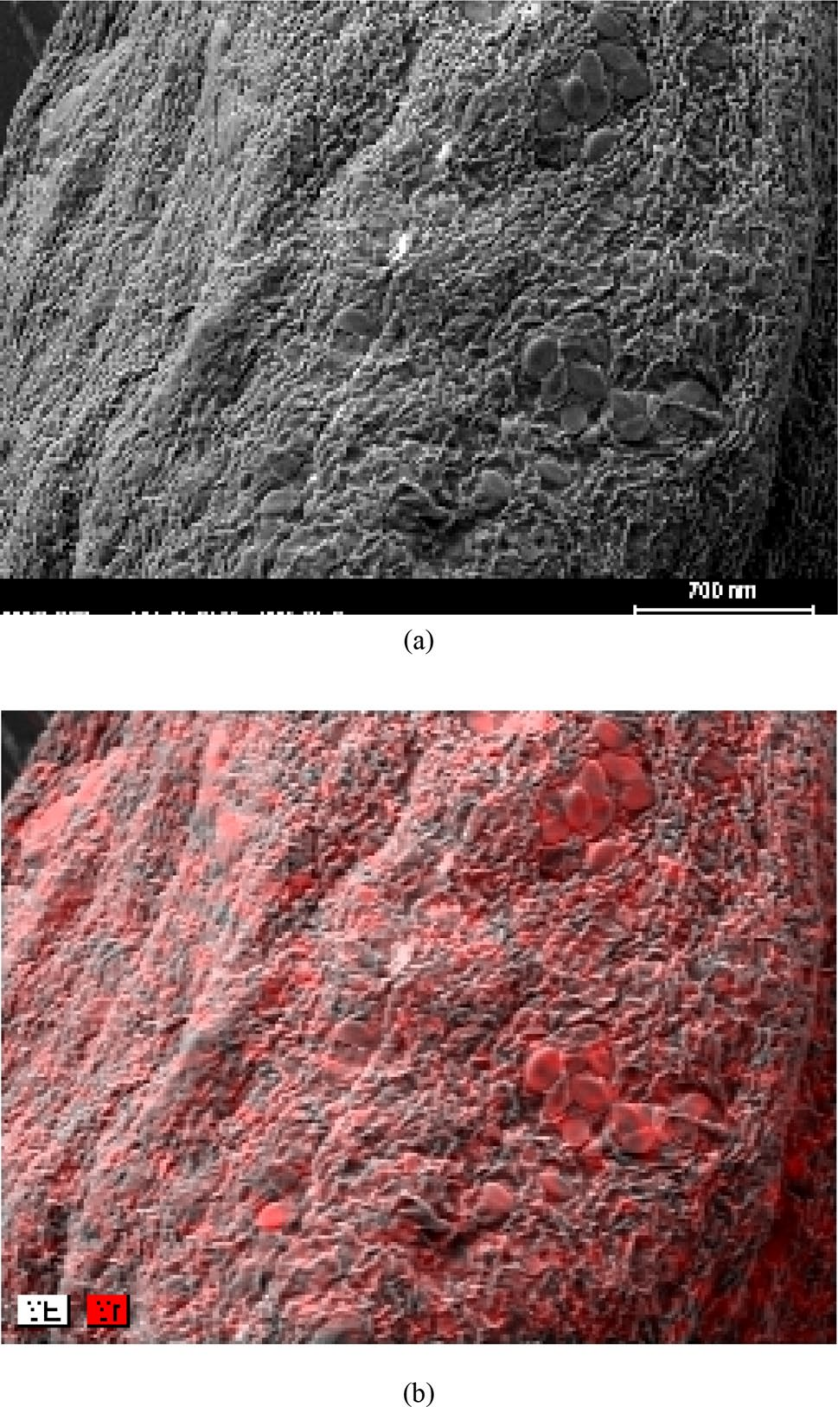
Element mapping of the surface of sargassum sp. earlier (**a**) and later (**b**) Sr^2+^ sorption at optimal conditions.

**Figure 5:**
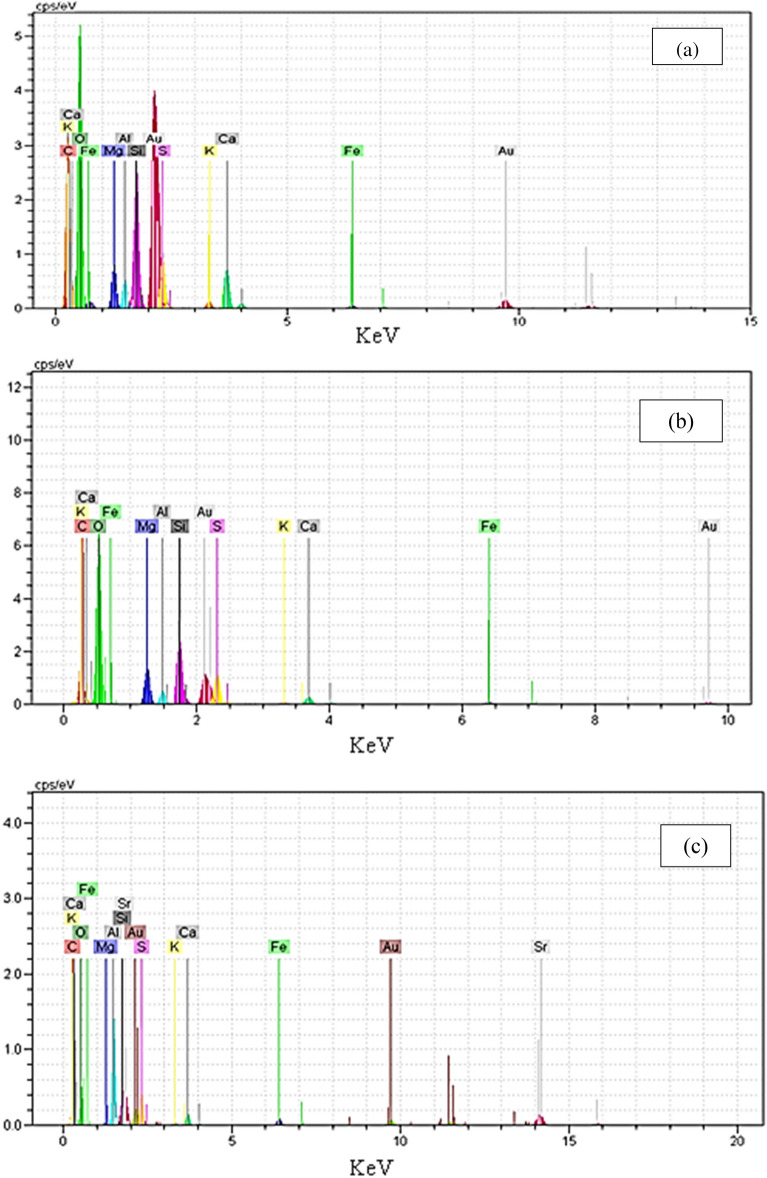
EDX analysis of sargassum sp. before (**a**) and after (**b**) Mg treatment, and after (**c**) strontium (II) sorption. Au peak comes from sample coating.

Assuming other variables are kept at their central values, the interplay between the type of biomass and initial strontium (II) concentration is illustrated in Fig. 3. The adsorption capacities of both untreated sargassum sp and treated were assessed for initial strontium (II) concentration ions at different concentrations and it was found that Mg(NO_3_)_2_ treatment enhanced the adsorption capacity. At the sorption of initial strontium (II) concentration, the adsorption capacity for untreated *sargassum sp* increased from 17.03 to 58.59 mg/g, while this change for treated *sargassum sp* was from 17.38 to 68.84 mg/g with the increasing of the initial concentration in 50–300 mg/l. Consistent with these results, magnesium nitrate enhances adsorption capacity by acting as an effective biosorbent surface modifier. As shown in Table 2, initial strontium (II) concentration was significantly influenced by the type of biomass (BE) (*p* = 0.003). There was a clear uniform distribution of Sr on the surface of the biosorbent when element mapping was performed (Fig. 4a,b). A spot profile analysis of EDX spectra shows an enhancement of magnesium peak intensity compared with the spectra of biomass before magnesium treatment (Fig. 5a,b) and confirm the existence of strontium (Fig. 5c), signifying cations of metal are bonded to algae surfaces by negatively charged functional groups. Mg-treated biomass, strontium-loaded biomass, and untreated *sargassum sp*. were tested using EDX spectrometers as indicated in Table 4. Based on the data presented in this study, it appears that the Sr^2+^ ions have substituted the Mg^2+^ ions from the surface of the biosorbent and that the ion exchange mechanism participates in strontium biosorption. Because of the complexity of biomaterials and other mechanisms, e.g. chelation of metals and coordination, cations could adsorb on biomass and surface complex at the same time, to differ the degrees, in accordance with the biosorbent and solution chemistry^62–64^.

**Table 4:** The amount of magnesium and strontium in the biomass.

Type of biomass	Element [wt. %]	
	Strontium	Magnesium
Mg-treated *Sargassum*	0.0	3.97
*sp.*Untreated *Sargassum sp.*	0.0	1.91
strontium-loaded *Sargassum sp.*	18.59	0.0

### 2D and 3D response plots

**Figure 6:**
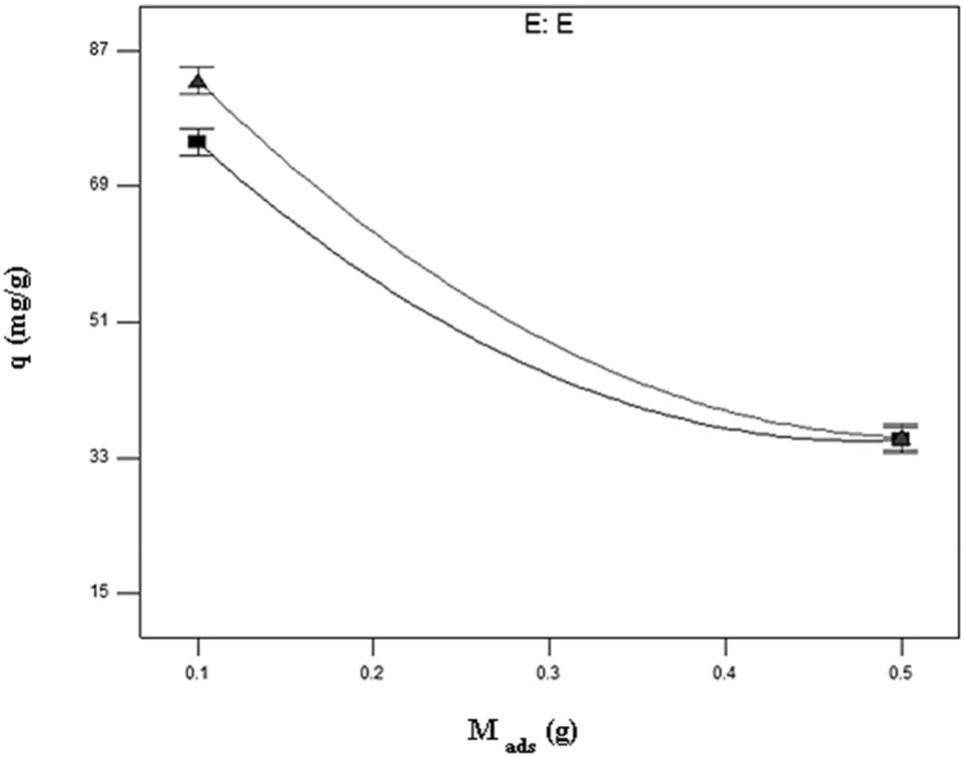
Two-factor interaction plot for biosorption capacity showing effects of biomass dose and type of biomass on the biosorption capacity for Mg-treated biomass holding other variables at their central values.

**Figure 7:**
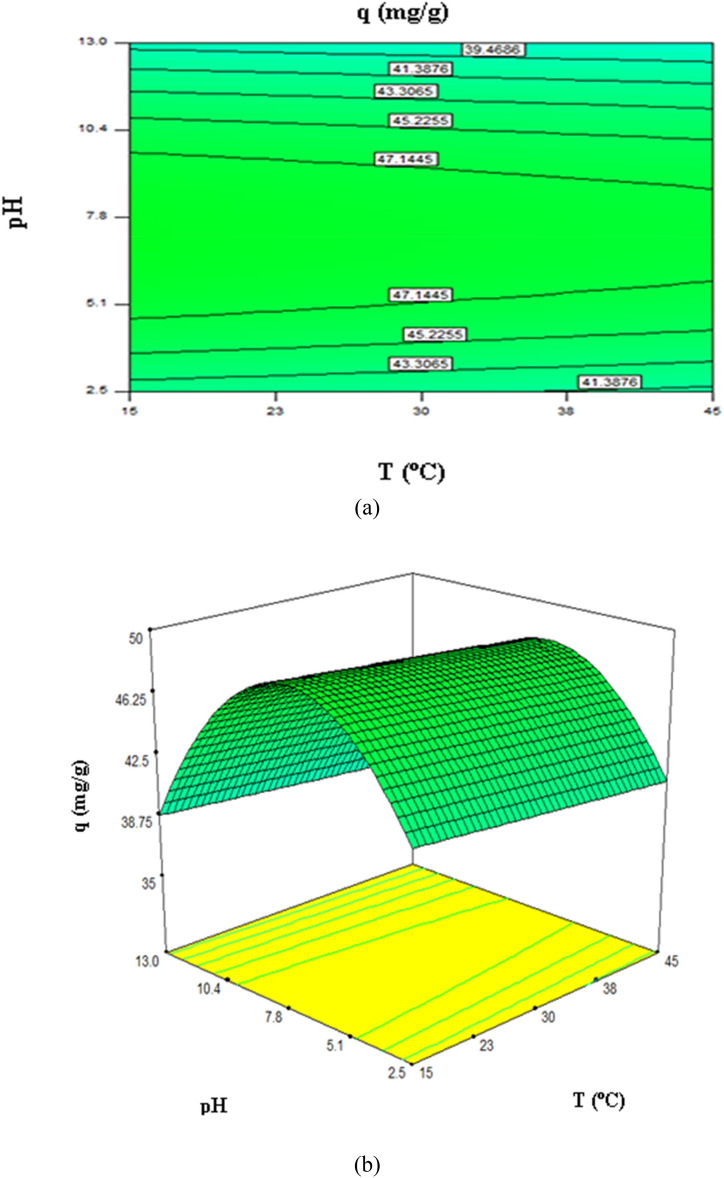
Three-dimensional plots presentation of the effects of pH and temperature (AD) on the strontium uptake of Mg-treated biomass holding other variables at their central values.

**Figure 8:**
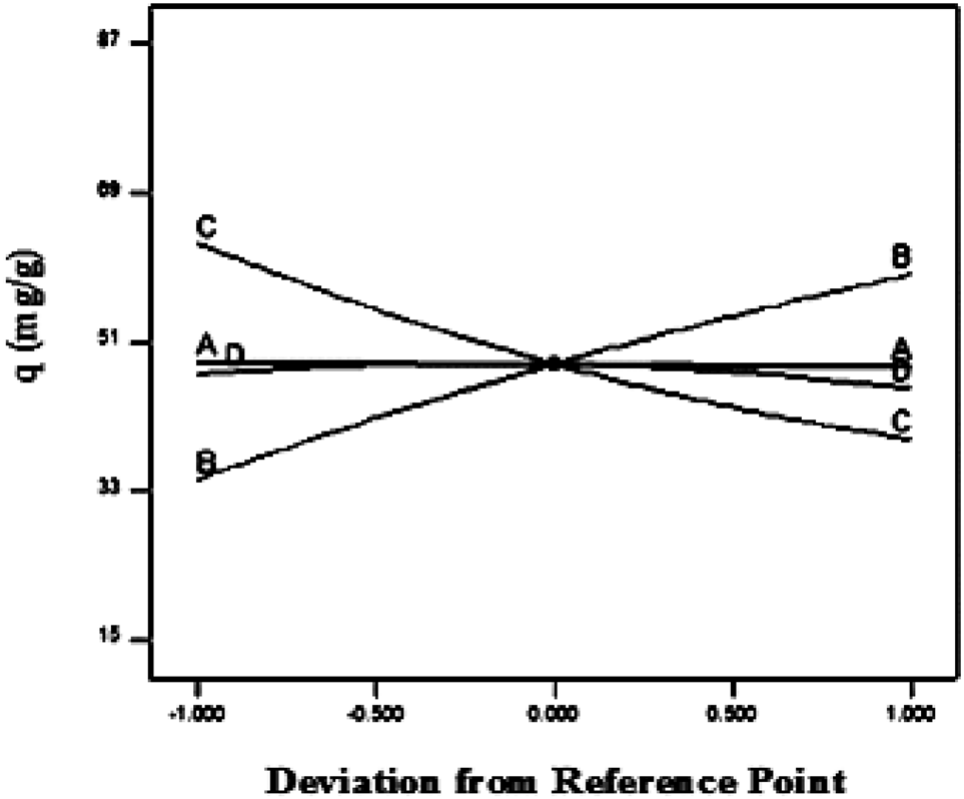
Perturbation plot for strontium (II) biosorption on Mg-treated sargassum sp.

As shown in Figs. 6, 7, 8, the relationship between parameters is plotted in two and three dimensions according to their respective response plots. During the plot generation, two factors varied while the other factors remained constant in center values.

Metal ion transfer to sorbents is predominantly driven by the initial metal concentration, with higher metal concentrations resulting in greater metal uptake^2,8,65^. Similarly, in the present study, Sr2 + concentration increased from 50 to 300 mg/l increasing biosorption capacity by about 49.46 mg/g (Fig. 3). Sargassum sp's active sites will be surrounded by more strontium ions if the solution contains more strontium ions, therefore biosorption will occur more efficiently.

The response plot shown in Fig. 6 indicates how biomass dosage affects Sr concentration at 175 ppm and pH at 7.8. The biosorption capacity decreased from 83.07 to 35.73 mg/g for the Sr^2+^ ions, as the biosorbent dosage was increased from 0.1 to 0.5 g in 100 mL solution. In the case of higher dosages of biosorbent, the capacity of uptake is lower. It would be related to the unsaturation of biosorption sites through the biosorption reaction as well as the particle interaction, for instance, aggregation resulting from high biosorbent concentration^66^.

As shown in Fig. 7, pH and temperature (AD) affect strontium adsorption at the constant initial concentration (175 mg/L) and dosage (0.3 g). As pH increases above pH 7.2, sargassum sp’s biosorption capacity decreases with pH. Figure 7 shows that strontium (II) uptake was not significantly affected by biosorption temperature. Based on the ANOVA results, the solution temperature was not statistically significant (*p*-value = 0.303). Many adsorbate-adsorbent systems have also shown similar results^8,67,68^.

Plotting the perturbation plot (Fig. 8) reveals how each factor affects Mg-treated biomass at the central point in the design space, compared. This plot is based on an initial strontium (II) ion concentration of 175 mg/L, a biosorbent dose of 0.3 g, a temperature of 30 °C, and an initial pH of 7.8. The steep slope in biosorption capacity versus initial strontium (II) ion concentration indicates that biosorption capacity is very sensitive to those factors (see Fig. 8). It is evident from the relatively flat line of temperature that it is insensitive to changes in that factor.

### Confirmatory experiment

A total of two experiments were conducted to validate the optimized model conditions based on values recommended by the models. The experimental results in Table 5 indicate good agreement between experimental results and the predicted values from the fitted correlations at a 95% confidence interval. The resulting experimental values matched the predicted values quite closely, proving that the model was valid. These conditions led to responses of 105.5 mg/g and 102.4 mg/g for biosorption capacity in the interval.

**Table 5:** Verification of the model at optimum condition.

Run	Response (maximized Sr^2+^ uptake)		95%C.I. low	95% C.I. high
	q_pre_ (mg/g)	q_exp_ (mg/g)	q (mg/g)	
1	101.722	102.4	97.79	105.66
2	101.722	105.5		

### Kinetic study

As part of this research, second-order and first-order reaction rate kinetic models were applied to explain strontium sorption on *sargassum sp*. Equations (5) and (6) are linear forms of pseudo-second-order (PSO) and pseudo-first-order (PFO) kinetic models, respectively:

5 $$\frac{t}{{q_{t} }} = \frac{1}{{k_{2} q_{eq}^{2} }} + \frac{1}{{q_{eq} }}t$$

6 $$\log (q_{eq} - q_{t} ) = \log q_{eq} - \frac{{k_{1} t}}{2.303}$$

where q_t_ (mg g^−1^) and q_eq_ are the amount of strontium adsorbed at any given time t (min) and at equilibrium, respectively. k_1_ (min^−1^) show the equilibrium rate constant of pseudo-first-order and k_2_ (g mg^-1^ min^-1^) show the equilibrium rate constant of second-order adsorption. To determine k1 and q_eq_, one has to plot the log (q_eq_–q_t_) versus time in a linear plot. On the other side, t/qt versus time can be used to determine k2 and q_eq_.

**Figure 9:**
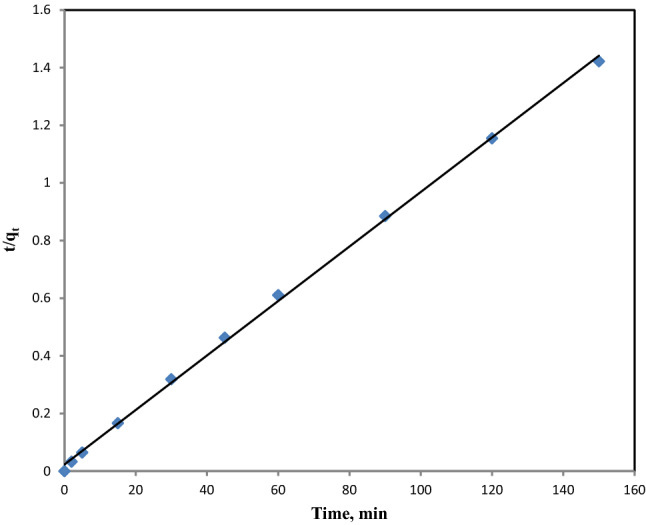
The linearized PSO biosorption dynamics of strontium.

Mg-treated biomass was subjected to two kinetics models to determine the biosorption of strontium ions at optimized conditions including C_0_ = 300 ppm, pH = 7.2, and Mads = 0.1 g. Results showed that the PFO model failed to explain all data obtained during contact time; therefore, coefficients with lower correlation were found (not presented). There have been reports of similar observations for a range of metals sorbing from aqueous solutions by biomass, proposing that it is a commonly observed feature in systems of sorption^65,69^. Because of the main limitation of the first-order equation of Lagergren, i.e. being normally appropriate for the initial rapid uptake, the sorption process takes about 20–30 min^65^. A plot of t/qt versus t for the PSO model can be seen in Fig. 9. Table 6 shows that theoretical q_e_(cal) values and experimental q_e_(exp) values are in good agreement. A correlation coefficient (R^2^) of 0.999 indicates that the present adsorption system is more well-defined by a PSO process. Because the PSO kinetic model provided the best fit, it can be expected that the rate-limiting step would be the chemisorption step^65,70^.

**Table 6:** Biosorption kinetics constants of strontium ions on Mg-treated biomass for the PSO model at optimized conditions (M_ads_ = 0.1 g/100 mL, pH = 7.2, C_0_ = 300 ppm and T = 28 °C).

q_e (exp)_ (mg/g)	k_2_ (g/mg min)	q_e (cal)_ (mg/g)	R^2^
105.5	0.0039	105.26	0.9991

### Isotherm at optimized condition

A comparison of Langmuir and Freundlich isotherms, classical sorption models, was performed to ascertain the relationship in the middle of adsorbed strontium ions on algal cells (q_eq_) and unadsorbed strontium ions in solution (C_eq_). Equations (7) and (8) give the linear form of Langmuir and Freundlich isotherms, respectively:

7 $$\frac{1}{{q_{eq} }} = \frac{1}{{bq_{m} C{}_{eq}}} + \frac{1}{{q_{m} }}$$

8 $$\log q_{eq} = \log k + \frac{1}{n}\log C_{eq}$$

where q_eq_ (mg/g) is the equilibrium adsorbed amount, q_m_ (mg/g) is the Langmuir constant, which represents the maximum adsorption capacity of monolayers, C_eq_ (mg/l) is the equilibrium concentration, and b (l/mg) is a measure of binding affinity based on the energy of adsorption^71,72^. A linear plot of 1/qeq versus 1/Ceq is used to determine the equilibrium parameters, i.e. q_m_ and b. The constants k and n define the amount of adsorption and its intensity, respectively. 1/n and k can be obtained from plots of log q_eq_ as a function of C_eq_.

Design-Expert software calculated the isotherm of strontium sorption on Mg-treated biomass at constant optimized conditions such as pH = 7.2 and M_ads_ = 0.1. Based on that data, the isotherms of strontium adsorption at 28 °C are provided in Table 7. Compared with Langmuir, the Freundlich model had better correlation coefficients (R^2^) for the adsorption equilibrium of strontium which implies the multilayer sorption of the strontium ions on the biosorbent^73^. As can be seen from Table 7, higher n values, i.e. more than unity, suggest satisfactory biosorption situations and the formation of stronger bonds between adsorbate and adsorbent^67,74^.

**Table 7:** Adsorption isotherm parameters for Sr(II) biosorption on Mg-treated *sargassum sp.*

T (ºC)^a^	Langmuir constants			Freundlich constants		
	q_m_ (mg/g)	b (l/mg)	R^2^	k	n	R^2^
28	87.719	0.686	0.85	42.628	6.397	0.95

^a^Has no effect on optimization results.

## Conclusion

In this work, Sargassum sp. studied strontium uptake in intact and chemically modified forms*.* In the experimental design, CCD was used for the RSM, which was verified to be an effective strategy for trying to the impact of operating conditions on the process. It was found that a reduced quadratic model was able to expect the biosorption capacity. Based on the results of the ANOVA, the experimental and model data were significantly in agreement. Optimal biosorption conditions included magnesium-treated biomass, pH of 7.2, 0.1 g/100 mL biosorbent dose, and 300 mg/l strontium (II) ion concentration. In optimum conditions, Mg-treated biomass had a biosorption capacity of 103.95 mg/g for strontium (II) ions. It has been found that the Freundlich model is the most suitable for fitting the results which imply the multilayer sorption of the strontium ions on the biosorbent. Finally, the pseudo-second-order kinetics model described strontium (II) biosorption onto algal biomass well.

## Data Availability

The datasets used and/or analyzed during the current study are available from the corresponding author on reasonable request.

## List of symbols

*b*: The Langmuir constant (l/mg)
*b*_*0*_*, b*_*i*_* and b*_*ij*_: Linear and quadratic interaction coefficients, *i* and *j* = 1–5
*C*: Metal ion concentration at any time (mg/l)
*C*_*0*_: Initial metal ion concentration(mg/l)
*C*_*eq*_: Residual metal ion concentration at equilibrium (mg/l)
*C.I.*: Confidence interval (−)
*C.V*.: Coefficient variation (−)
*k*: Adsorption capacity (−)
*n*: Biosorption intensity (−)
*q*: Amount of biosorbed metal per g of biosorbentat any time (mg/g)
*q*_*eq*_: The amount of biosorbent metal per unit weight of biosorbent at equilibrium(mg/g)
*q*_*exp*_: Experimental amount of biosorbent metal per unit weight of biosorbent at equilibrium (mg/g)
*q*_*m*_: Maximum capacity of biosorbent
*q*_*pre*_: The predicted amount of biosorbent metal per unit weight of biosorbent at equilibrium by software(mg/g)
*R*^*2*^: Correlation coefficient
*R*^*2*^_*adj*_: Adjusted correlation coefficient
*M*_*ads*_: Amount of biosorbent (g)
*t*: Time (min)
*x*_*i*_: The independent variable, i = 1–5
*y*: Response (−)
*V*: The volume of the solution (l)
*T*: Solution temperature (°C)
